# Label Free Detection of L-Glutamate Using Microfluidic Based Thermal Biosensor

**DOI:** 10.3390/bioengineering2010002

**Published:** 2015-01-12

**Authors:** Varun Lingaiah Kopparthy, Siva Mahesh Tangutooru, Eric J. Guilbeau

**Affiliations:** 1 The Center for Biomedical Engineering and Rehabilitation Science, Louisiana Tech University, Ruston, LA 71272, USA; E-Mail: vlk002@latech.edu; 2 Department of Mechanical & Industrial Engineering, Qatar University, P.O. Box 2713, Doha, Qatar; E-Mail: sivamahesht@qu.edu.qa

**Keywords:** thermoelectric, label-free, biosensor, L-glutamate, L-glutamate oxidase, layer-by-layer self-assembly

## Abstract

A thermoelectric biosensor for the detection of L-glutamate concentration was developed. The thermoelectric sensor is integrated into a micro-calorimeter which measures the heat produced by biochemical reactions. The device contains a single flow channel that is 120 µm high and 10 mm wide with two fluid inlets and one fluid outlet. An antimony-bismuth (Sb-Bi) thermopile with high common mode rejection ratio is attached to the lower channel wall and measures the dynamic changes in the temperature when L-glutamate undergoes oxidative deamination in the presence of glutamate oxidase (GLOD). The thermopile has a Seebeck coefficient of ~7 µV·(m·K)^−1^. The device geometry, together with hydrodynamic focusing, eliminates the need of extensive temperature control. Layer-by-layer assembly is used to immobilize GLOD on the surface of glass coverslips by alternate electrostatic adsorption of polyelectrolyte and GLOD. The impulse injection mode using a 6-port *injection* valve minimizes sample volume to 5 µL. The sensitivity of the sensor for glutamate is 17.9 nVs·mM^−1^ in the linear range of 0–54 mM with an R^2^ value of 0.9873. The lowest detection limit of the sensor for glutamate is 5.3 mM.

## 1. Introduction

The amino acid L-glutamate is the major excitatory neurotransmitter, which plays a key role in the human central nervous system and metabolism [1]. Glutamate is involved in many of the major brain functions such as memory, cognition and learning. Cellular mechanisms underlying learning and memory such as long-term depression and long term potentiation are also associated with the glutamatergic synapses connectivity strength [2]. The extent of signal stimulation is determined by the glutamate concentration in the extracellular fluid [1]. The brain contains more glutamate than any other amino acid. The concentrations of glutamate in the extracellular fluid of the brain and cerebrospinal fluid (CSF) are about 3–4 µM and 10 µM, respectively [3,4,5]. The concentration of L-glutamate in the intracellular fluid (cytosol) is very high, at around 1–10 mM. The highest glutamate concentrations are found in nerve terminals and the concentration inside synaptic vesicles may be as high as 100 mM. The concentration of glutamate is maintained at a low value because high concentrations of glutamate in the extracellular fluid increase the signal stimulations, which lead to neurological disorders [1]. Glutamate is also directly involved in neurological disorders such as stroke, and neurodegenerative disorders such as epilepsy, Parkinson’s disease, and Alzheimer’s disease [1,6]. For these reasons, monitoring of L-glutamate concentration is important in understanding neurobiology and neuropathology.

### 1.1. L-Glutamate Sensors: State of the Art and Limitations

Many sensors have been developed to detect the concentration of glutamate *in vitro* [7,8,9,10,11,12,13,14,15] and *in vivo* [16,17,18,19]. Some sensors have also been implanted into animals and used to measure L-glutamate in the brain [20,21,22,23]. These sensors were based on different sensing techniques such as electrochemical [8,9] and optical detection [24,25,26]. Although these methods were established and standardized, there are many disadvantages such as difficult fabrication techniques, complex detection systems, instability, power consumption and cost. Therefore, a low cost, highly stable, and easily fabricated glutamate sensor is needed to better understand the role of glutamate in pathophysiology. 

*In vivo* detection of L-glutamate using electrochemical method has a major disadvantage for sensitivity to L-glutamate in the presence of electroactive interferents in the extracellular environment [27]. Current electrochemical sensors have many limitations such as interference from reducing agents such as ascorbic acid (AA), uric acid (UA), dopamine (DA), cysteine on the electrodes and biofouling due to unspecific attachment of bioactive species onto the electrodes. Protective layers [28] and additional enzymes such as ascorbic acid oxidase [18] were used to reduce the effect of interferent signals and reducing agents. To understand glutamate behavior in the brain, there is a great need for a sensor that can detect glutamate release from synaptic origin [27]. Electrochemical detection of glutamate release from synaptic clefts is still challenging due to the size imitations of the electrodes [27]. The proposed study in this article is based on calorimetric sensing; L-glutamate undergoes oxidative deamination in the presence of GLOD releasing heat, which is detected by the thermopile sensor. Since the thermoelectric sensor is based on heat detection, this study is label-free and eliminates several challenges faced by the current detection methods. Thermoelectric detection of L-glutamate could potentially be an alternative platform to understand the dynamics of L-glutamate release. With increased sensitivities, the proposed method can potentially be used to detect real time glutamate release from cultured nerve cells. 

### 1.2. Calorimetric Biosensing

Calorimetric biosensors have been used to detect various analytes such as glucose [29,30,31] and urea [32]. Calorimetric approach takes advantage of the universal nature of heat power production of chemical reactions. These sensors have many advantages over amperometric and optical detection methods such as label-free detection, relatively easy fabrication and less complex detection systems using voltage detectors. Thermopiles are widely used in calorimetric biosensors due to their advantages such as high common mode thermal noise rejection ratio and can be used with miniaturized devices. Reference junctions of the thermopiles in calorimetric biosensors are controlled either by a constant heat source, heat sink or by vacuum encapsulation. Controlling reference temperature adds complexity to the system and requires additional components. Utilizing the thermopiles’ high common mode rejection of thermal signals, in this paper we present a label-free, highly-sensitive, interferent-free, microfluidic thermoelectric biosensor for the detection of L-glutamate without need to control reference junction temperature. The sensor, which detects the heat of the reaction, is a thermopile, which produces a self-generating signal. A microfluidic device is used to initiate and isolate the chemical reaction and the enzyme is immobilized using layer by layer self-assembly. The device is fabricated using an inexpensive fabrication technique. 

## 2. Experimental

### 2.1. Materials and Chemicals

Glass microscope slides (thickness—1.2 mm), glass microscope coverslips (thickness—0.17 mm) and round coverglass (5 mm diameter and 1.5 mm thick) were obtained from Electron Microscopy Sciences (Hatfield, PA, USA). *Kapton*^®^ tape (thickness—100 µm) was purchased from kaptontape.com (CA) and *kapton*^®^ tape with dual side adhesive (polyimide film, thickness—120 µm) from Dupont (Wilmington, DE, USA). Cutting plotter was obtained from Graphtec America Inc., (Santa Ana, CA, USA). Syringe pumps (Model “11” Plus) were obtained from Harvard Apparatus, (Holliston, MA, USA). 1/16 inch outer diameter ports, 6-port *Injection* valve and 0.01 inch internal diameter Teflon ETFE tubing were obtained from Upchurch Scientific (Oak Harbor, WA, USA). Agilent Model 34420A nano-voltmeter was obtained from Agilent, Inc., (Loveland, CO, USA) and National Instruments Signal Express from National Instruments Corporation, (Austin, TX, USA).

L-glutamate, L-glutamate oxidase (GLOD), *polyethyleneimine* (PEI) and *Polystyrenesulfonate* (PSS) were purchased from Sigma-Aldrich (St. Louis, MO, USA). Amplex^®^ Red L-glutamate oxidase assay kit is obtained from Invitrogen Corporation (Carlsbad, CA, USA).

### 2.2. Device Fabrication

**Figure 1:**
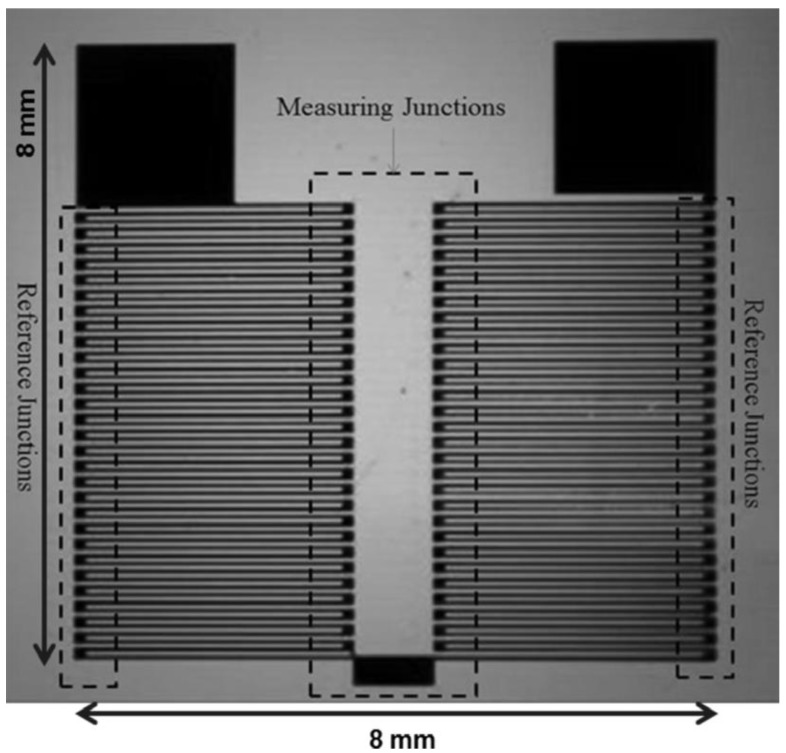
Thermopile (8 mm × 8 mm) fabricated on 100 µm *kapton*^®^ support showing measuring and reference junctions.

**Figure 2:**
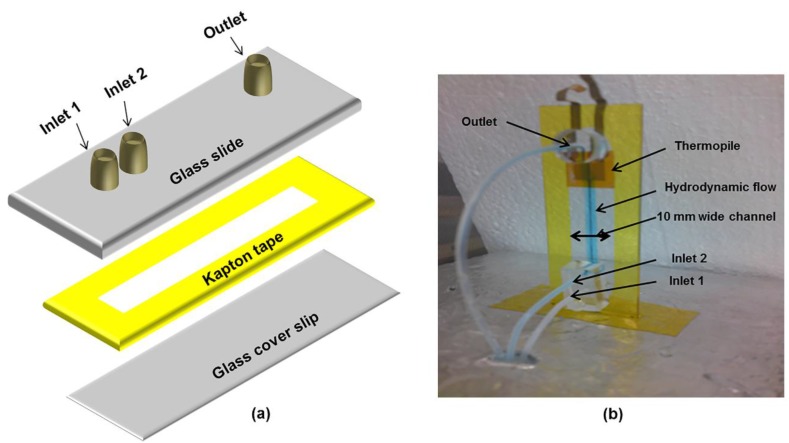
Thermoelectric microfluidic L-glutamate sensor. (**a**) Schematic showing microfluidic device fabrication. (**b**) Microfluidic device showing hydrodynamic flow and thermopile attached on the lower channel wall of the microfluidic device. The dimensions of the components are glass slide—75 × 25 × 1.2 mm, glass coverslip—75 × 25 × 0.17 mm, and channel—60 × 10 × 0.12 mm. Volume of the microfluidic channel is 72 µL.

The thermopile is fabricated on *kapton*^®^ tape (100 µm) using the procedure explained in our earlier paper using metal masks in a thermal evaporator to form an antimony-bismuth thin film thermopile [33]. Briefly, a metal mask with patterned design was used to deposit 0.8 µm thick bismuth film, and complimentary design was used to deposit 1.2 µm thick antimony films on to a 100 µm *kapton*^®^ tape to form a thermopile (Figure 1). Thermopile is 8 mm wide, 8mm long and has a 60 thermocouple junctions with a Seebeck coefficient of ~7 µV·(m·K)^−1^. The characteristics of the fabricated thermopile were discussed in our earlier paper [33]. Inlet and outlet holes are drilled on the microscope glass slide along the center line and 1/16 outer diameter ports are attached over the holes using an adhesive ring and baking it in the oven at 170 °C for 45 min. Dual side adhesive *kapton*^®^ tape (120 µm) is cut according to the rectangular channel design (60 mm long and 10 mm wide) using a cutting plotter and is sandwiched between the prepared microscopic glass slide and glass coverslip to form a microfluidic device (Figure 2a). The fabricated thermopile is attached beneath the outer channel wall of the microfluidic device (Figure 2b). 

### 2.3. Enzyme Immobilization

**Figure 3:**
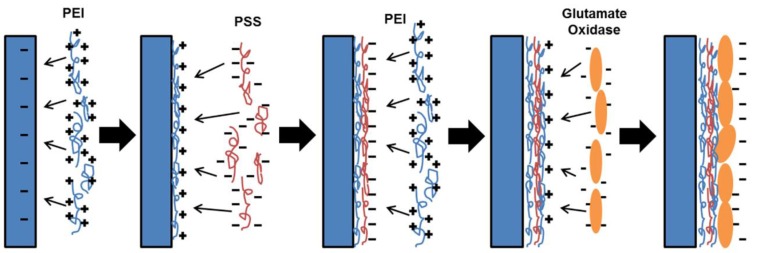
Layer-by-layer assembly of glutamate oxidase (GLOD) on a substrate. Electrostatic adsorption and charge resaturation occurs on the substrate to form alternate layers of polyelectrolyte and GLOD films.

Procedure for immobilizing proteins and enzymes using layer by layer assembly was illustrated by Lvov *et al*. [34]. Layer by layer assembly was adopted to immobilize GLOD on glass coverslips and this procedure is explained here. GLOD is mixed with reaction buffer from an Amplex^®^ red assay kit to obtain 5 U·mL^−1^ solution. 100 µL of PEI (50% (W/V)) is mixed with 10 mL of deionized (DI) water to obtain a concentration of 5 mg·mL^−1^. 25 mg of PSS is dissolved in 5 mL of DI water to obtain a concentration of 5 mg·mL^−1^. The microscope glass coverslip (negatively charged) is immersed into PEI (positively charged) solution for 5 min. Then the coverslip is rinsed in water for 1 min and dried under nitrogen. The PEI coated coverslip is immersed in PSS (negatively charged) solution for 15 min, rinsed in water for 1 min and dried under nitrogen. Now the coverslip is immersed in PEI solution following similar procedure to produce uniform PEI layer which is positively charged. After this procedure, the coverslip is immersed in L-glutamate (negatively charged) solution for 15 min, rinsed in water for 1 min and dried under nitrogen. This procedure may be repeated to form multiple layers of GLOD. A schematic of the layer-by-layer adsorption procedure is shown in Figure 3. Initial layers of PEI and PSS on glass coverslip act as precursor layers to produce uniform and strong electrostatic charge. Although the glass coverslip is negatively charged, the surface of a plain glass coverslip is not completely uniform charge. To ensure proper GLOD immobilization, precursor layers are recommended.

### 2.4. Measurement System

**Figure 4:**
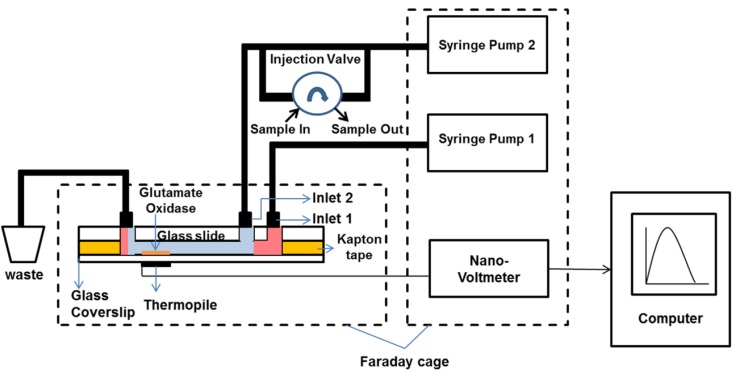
Experimental setup (schematic) of the thermoelectric detection system. Sample loop size used in the injection valve is 5 µL.

Syringe pumps are used to introduce DI water continuously into inlet 1 and inlet 2 at a flow rate of 100 µL·min^−1^ and 25 µL·min^−1^, respectively. An inlet 2 flow in the device is hydrodynamically focused due to the geometry of the microfluidic device and viscous forces dominate the inertial forces, since the channel height is 100 µm. An injection valve connected to inlet 2 is used to introduce various concentrations of L-glutamate samples into the microfluidic device. To enable the reaction only on the measuring junctions of the thermopile, hydrodynamic focusing is used to focus the flow only on to the measuring junctions, this produces temperature change between the measuring and reference junctions. Fluid flows in the device from inlet 1 and with inlet 2 hydrodynamically focused will eliminate the need to control the thermopile reference junction temperature. Schematic of the experimental setup is shown in Figure 4. When the L-glutamate sample reaches the thermopile, it reacts in the presence of GLOD releasing heat (30 kJ·mol^−1^) (Equation (1)). The enthalpy of the reaction was calculated from the heats of formations of products and reactants [35]. This heat is detected by the thermopile producing proportional voltage, which is detected by the nano-voltmeter and recorded using National Instruments Signal Express in a digital computer. 

(1) $$L-glutamate+O_{2}+H_{2}O\overset{glutamate\;oxidase}{\to }\alpha -ketoglutarate+NH_{3}+H_{2}O_{2}+30\;kJ/mol$$

## 3. Results and Discussion

### 3.1. Activity and Storage Stability of Immobilized GLOD

**Figure 5:**
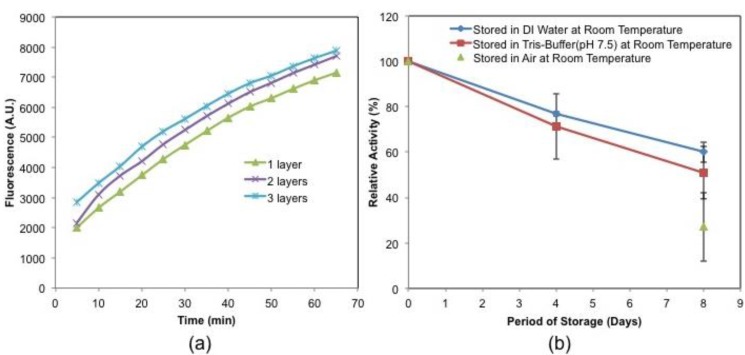
(**a**) Activity of the immobilized GLOD layers using Amplex^®^ Red reagent based assay. Fluorescence was measured for every 5 min using a fluorescence microplate reader using excitation at 530 ± 12.5 nm and the fluorescence detection at 590 ± 17.5 nm. Activity of one layer (*polyethyleneimine* (PEI)/GLOD), two layers ((PEI/GLOD)_2_) and three layers ((PEI/GLOD)_3_) of GLOD was studied. (**b**) Stability of immobilized GLOD film when stored in de-ionized (DI) water, Tris-Buffer (pH 7.5) and air at room temperature. GLOD immobilized coverslips were tested for a period of 8 days. Error bars represent standard error when n = 3.

Once the GLOD is immobilized on glass coverslips using layer by layer assembly, it is crucial to test the activity of GLOD to ensure L-glutamate reaction in the fabricated microfluidic device. To test the activity of the immobilized GLOD, round glass coverslips were used to immobilize one layer, two layers, and three layers of GLOD. These glass coverslips were used to test the activity using Amplex^®^ Red glutamate oxidase fluorescence assay kit. The activity tests were performed following the instructions described in the assay procedure. The enzyme activity of these layers was then detected by measuring the fluorescence of hydrogen peroxide, which is also a product in the L-glutamate reaction (Equation (1)), with Amplex^®^ red reagent using excitation at 530 ± 12.5 nm and the fluorescence detection at 590 ± 17.5 nm. The activity of the GLOD increased with the number of immobilized GLOD layers. Figure 5a shows the activity of immobilized GLOD layers. 

Long term use of thermoelectric L-glutamate sensor depends on the stability of immobilized GLOD films. To test the stability of GLOD films, round glass coverslips with immobilized GLOD were stored in three different conditions such as DI water, Tris-Buffer (pH 7.5) and air (open environment) at room temperature. The activity of these stored GLOD films were observed for a period of 8 days. Figure 5b shows the storage stability of immobilized GLOD in air, water and buffer. The activity of the GLOD films were decreased ~40%, ~49% and ~73% when stored in de-ionized (DI) water, Tris-Buffer (pH 7.5) and air respectively, when stored at room temperature. The stability of GLOD films stored in DI water and Tris buffer were affected possibly due to the testing of the films on day 4. 

Activity of the enzyme increases with an increase in the number of immobilized GLOD layers (Figure 5a). The increase in the activity for two and three layers is not significantly high compared to one layer of GLOD. This is possibly due to the uneven distribution of multiple GLOD layers on coverslips caused by ineffective charge resaturation on the first layer of GLOD, which would result in a varying GLOD concentration. Another reason would be the diffusion of the L-glutamate through the films or inactivation of the sandwich enzymes. The fluorescence intensity value saturated after 60 min for GLOD layers (Figure 5a). GLOD activity decreased (Figure 5b) significantly when stored in air at room temperature possibly due to GLOD became inactive when stored in air. When stored in DI water, the activity is only decreased by 40% over a period of 8 days. Since DI water is continuously flowing in the microfluidic device, the thermoelectric L-glutamate sensor can be used for up to 8 days with only 40% decrease in the sensitivity. For long term use of the sensor, additional studies are needed to be performed to obtain better storage option for higher sensitivity. 

Layer-by-layer adsorption was used in this study for GLOD immobilization due to its advantages such as layer growth in nanometers, suitable for microfluidic applications, simplicity, and inexpensive. Other methods of immobilization such as glutaraldehyde [16] crosslinking, and avidin [36] might be useful to investigate to increase loading and stability of the GLOD. Layer-by-layer adsorption was chosen in this study, because, glutaraldehyde immobilization is generally viscous and forms a thick layer (might obstruct the flow in the microfluidic device, since the channel height is 100 µm) and avidin chemistry involves complex process and is time consuming.

### 3.2. L-Glutamate Detection

**Figure 6:**
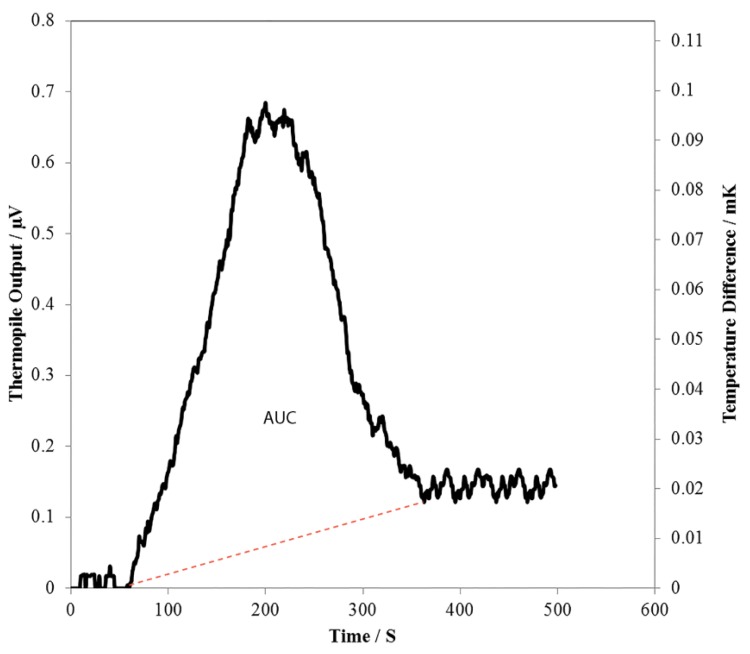
Thermopile output following the injection of 54 mM L-glutamate sample. Sample size used is 5 µL. Secondary ordinate axis represents the temperature rise (calculated from Seebeck coefficient) detected by the thermopile. The dotted line indicated in red shown in the figure is used to determine the area under the curve (AUC).

**Figure 7:**
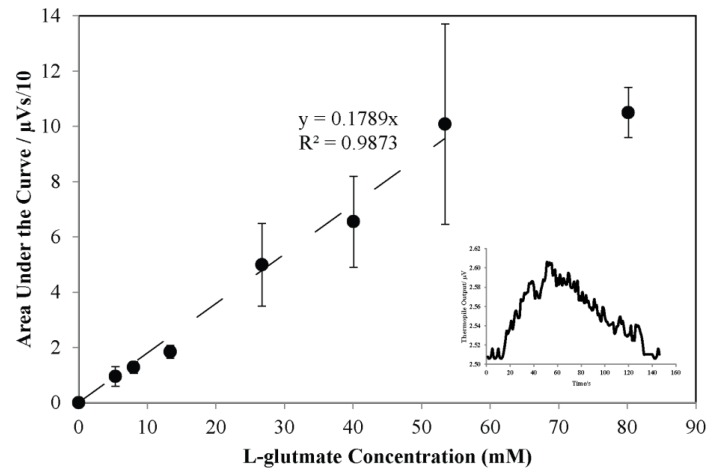
Integrated thermopile output plotted as a function of L-glutamate concentration. One layer of GLOD was immobilized. Concentrations of L-glutamate samples range from 0–80.2 mM. Dashed line is the linear fit indicating the linear range from 0–54 mM for immobilized GLOD (PEI/GLOD). The sensor has a lower detection limit of 5.3 mM. Error bars represent the standard error when n=4. Inset: thermopile output at the lower detection limit (5.3 mM).

Glutamate solutions of concentrations ranging from 0–80 mM were introduced using the 5 µL sample loop into the microfluidic device. The thermopile typically reads a stable baseline because DI water is continuously injected into inlet 1 and 2. When a glutamate sample (5 µL) is loaded into the injection valve and introduced into the inlet 2 flow stream, the sample reaches the thermopile in the microfluidic device. Thermopile response increases due to the heat released on the measuring junctions by the L-glutamate reaction in the presence of immobilized GLOD and slowly comes back to the baseline when the sample passes past the thermopile. Figure 6 shows the typical response of the thermopile following the injection of the glutamate sample. Area under the curve is a measure of total heat detected by the thermopile. Flow rates of 100 µL·min^−1^ at inlet 1 and 25 µL·min^−1^ at inlet 2 are used. One layer (PEI/GLOD) of GLOD is immobilized on the bottom channel wall of the microfluidic device. Figure 7 shows the response of the thermopile for various glutamate concentrations. The response is linear (Figure 7) in the range of 0–54 mM L-glutamate concentration. The response of the thermopile (Figure 7) saturates when the concentration of L-glutamate is greater than 54 mM, because the reaction is limited by oxygen concentration. The sensitivity of the sensor is 17.9 nVs·mM^−1^ in the linear range of 0–54 mM. The sensor has a lower detection limit of 5.3 mM, below which the sensor showed no response to L-glutamate concentrations. 

The response of the thermopile increases linearly for the injection of glutamate concentrations ranging from 0–54 mM. The response saturates after 54 mM because oxygen limits the reaction. Injection of oxygen saturated glutamate samples might increase the response linearly over broader range of L-glutamate concentrations. The variation in the standard error of the response is high, possibly due to the flow fluctuations caused by the syringe pumps. Since the enzyme is immobilized over the entire area of the thermopile, the effect of flow fluctuations on the thermopile responses are due to the change in the width of the hydrodynamic focusing causing variations in the reaction site area and the diffusion of glutamate. The thermopile response increases with an increase in glutamate concentration for one layer of immobilized GLOD (PEI/GLOD). The response increases linearly from 0–54 mM. Introducing oxygen saturated L-glutamate samples can increase the linear range, since the L-glutamate reaction is limited by oxygen availability.

Although the thermoelectric sensor has a wide linear range, very low concentrations of L-glutamate were not detected. Detecting very low concentrations in the physiological L-glutamate range is required for potential use of this sensor to detect dynamic L-glutamate release from nerve cells. Optimizing the immobilization procedure, increasing the diffusion of the sample and increasing the sensitivity by co-immobilizing enzymes like catalase to convert hydrogen peroxide (product of the L-glutamate reaction) to water and oxygen, releasing additional heat or glutamate dehydrogenase to recycle the oxoglutarate formed into L-glutamate thus amplifying the reaction, could possibly increase the sensitivity of the sensor. The sensor is based on heat detection; attempts to decrease the thermal resistance between the reaction site and the thermopile will significantly increase the sensitivity and performance of the sensor. 

### 3.3. Potential Uses of the Thermoelectric L-Glutamate Sensor

**Figure 8:**
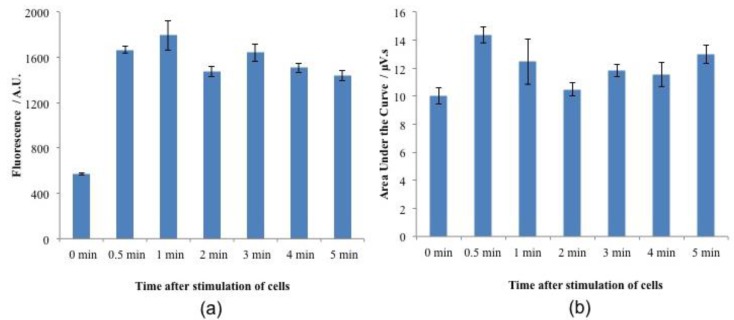
L-glutamate release from brain tumor cell line CRL 2303 media samples following potassium chloride (KCL) stimulation. The cells were plated in a petri dish at a density of 200,000 cells per mL. After 24 h, the cells were stimulated using 50 mM KCl. The samples of the cell media were collected in regular time intervals after treatment and analyzed using (**a**) fluorescence spectrophotometer using Amplex red assay, (**b**) the microfluidic thermoelectric L-glutamate sensor. Error bars represent the standard error when n = 3.

We have demonstrated that the thermoelectric method is an alternative platform for detecting L-glutamate with benefits over existing methods. The developed sensor was also used to detect extracellular L-glutamate concentrations from nerve cell cultures. Gliobastoma cells (cell line CRL 2303) were externally cultured in a petri dish according to the vendor recommendations. These cultured nerve cells were stimulated with 50 mM potassium chloride (KCl). Cell media samples were collected at regular intervals and these samples were injected into the thermoelectric L-glutamate sensor to detect the glutamate released by the nerve cell cultures following KCl stimulation. L-glutamate release was also measured using fluorescence spectrophotometer. Figure 8a shows the response of the fluorescence spectrophotometer using Amplex red assay. Figure 8b shows the response of the thermoelectric L-glutamate sensor following KCl stimulation. 

The fluorescence reading at zero min (before KCl stimulation) was non-zero because of the presence of L-glutamate in the cell culture media. The L-glutamate levels increased in the media collected at 30 s after stimulation of KCl. There was no significant uptake of L-glutamate until five minutes. This indicates that the cells might require more than five minutes to uptake the released L-glutamate. L-glutamate release from externally cultured brain tumor cells that were grown in a petri dish was also measured using the thermoelectric sensor. In these experiments, media samples were taken from the petri dish and injected into the microfluidic L-glutamate senor using the 6-port *injection* valve. These results were compared with the fluorescent spectrophotometer response as shown in Figure 8. In these experiments, samples were collected from the petri dish after regular intervals of time for five minutes after the stimulation with 50 mM KCl. 

The proposed sensor is based on heat detection, so the interference from heat signals must be considered while calibrating the sensor for biochemical process. In the future, with increased sensor sensitivities, L-glutamate dynamics can be studied by immobilizing nerve cell cultures directly over the thermal sensor inside the microfluidic device. Since the cultured nerve cells are immobilized over the thermopile area, interference of many biochemical processes releasing heat in the cells must be considered. Calibration of the sensor without immobilized glutamate oxidase and with immobilized glutamate oxidase must be performed. By subtracting the signal recorded without glutamate oxidase from the sensor with glutamate oxidase the response to glutamate can be analyzed. 

## 4. Conclusions

A thermoelectric biosensor for the detection L-glutamate was successfully fabricated. A microfluidic calorimeter was fabricated using an inexpensive and easy fabrication process with an integrated thermopile. The device was capable of handling small sample volumes. The sensor can be used to study the sensor response for L-glutamate released from the cultured nerve cells. Since the thermoelectric sensor developed is based on heat detection, the proposed method is label free and has potential to be an alternative platform for understanding the dynamics of neurotransmitter, L-glutamate.

## References

[1] Danbolt N.C. (2001). Glutamate uptake. Prog. Neurobiol..

[2] Kandel E.R. (2001). The molecular biology of memory storage: A dialogue between genes and synapses. Science.

[3] Hamberger A., Berthold C.-H., Karlsson B., Lehmann A., Nystrom B. (1983). Extracellular GABA, glutamate and glutamine *in vivo* perfusion-dialysis of rabbit hippocampus. Neurol. Neurobiol..

[4] Hamberger A., NystrÃm B. (1984). Extra-and intracellular amino acids in the hippocampus during development of hepatic encephalopathy. Neurochem. Res..

[5] Lehmann A., Isacsson H., Hamberger A. (1983). Effects of *in vivo* administration of kainic acid on the extracellular amino acid pool in the rabbit hippocampus. J. Neurochem..

[6] Nedergaard M., Takano T., Hansen A.J. (2002). Opinion: Beyond the role of glutamate as a neurotransmitter. Nat. Rev. Neurosci..

[7] Niwa O., Horiuchi T., Kurita R., Tabei H., Torimitsu K. (1998). Microfabricated on-line sensor for continuous monitoring of L-glutamate. Anal. Sci..

[8] Niwa O., Kurita R., Horiuchi T., Torimitsu K. (1999). Continuous monitoring of L-glutamate released from cultured rat nerve cells with a microfabricated on-line sensor at a slow flow rate. Electroanalysis.

[9] Niwa O., Horiuchi T., Torimitsu K. (1997). Continuous monitoring of L-glutamate released from cultured nerve cells by an online sensor coupled with micro-capillary sampling. Biosens. Bioelectron..

[10] Niwa O., Torimitsu K., Morita M., Osborne P., Yamamoto K. (1996). Concentration of extracellular L-glutamate released from cultured nerve cells measured with a small-volume online sensor. Anal. Chem..

[11] Castillo J., Blöchl A., Dennison S., Schuhmann W., Csöregi E. (2005). Glutamate detection from nerve cells using a planar electrodes array integrated in a microtiter plate. Biosens. Bioelectron..

[12] Pasco N., Jeffries C., Davies Q., Downard A.J., Roddick-Lanzilotta A.D., Gorton L. (1999). Characterisation of a thermophilic L-glutamate dehydrogenase biosensor for amperometric determination of L-glutamate by flow injection analysis. Biosens. Bioelectron..

[13] Basu A.K., Chattopadhyay P., Roychudhuri U., Chakraborty R. (2006). A biosensor based on co-immobilized L-glutamate oxidase and L-glutamate dehydrogenase for analysis of monosodium glutamate in food. Biosens. Bioelectron..

[14] Tseng T.T.C., Yao J., Chan W.-C. (2013). Selective enzyme immobilization on arrayed microelectrodes for the application of sensing neurotransmitters. Biochem. Eng. J..

[15] Tseng T.T.C., Monbouquette H.G. (2012). Implantable microprobe with arrayed microsensors for combined amperometric monitoring of the neurotransmitters, glutamate and dopamine. J. Electroanal. Chem..

[16] Hu Y., Mitchell K.M., Albahadily F.N., Michaelis E.K., Wilson G.S. (1994). Direct measurement of glutamate release in the brain using a dual enzyme-based electrochemical sensor. Brain Res..

[17] Day B.K., Pomerleau F., Burmeister J.J., Huettl P., Gerhardt G.A. (2006). Microelectrode array studies of basal and potassium-evoked release of L-glutamate in the anesthetized rat brain. J. Neurochem..

[18] Kulagina N.V., Shankar L., Michael A.C. (1999). Monitoring glutamate and ascorbate in the extracellular space of brain tissue with electrochemical microsensors. Anal. Chem..

[19] Wassum K.M., Tolosa V.M., Tseng T.C., Balleine B.W., Monbouquette H.G., Maidment N.T. (2012). Transient extracellular glutamate events in the basolateral amygdala track reward-seeking actions. J. Neurosci..

[20] Morita H., Abe C., Awazu C., Tanaka K. (2007). Long-term hypergravity induces plastic alterations in vestibulo-cardiovascular reflex in conscious rats. Neurosci. Lett..

[21] Cairns B.E., Dong X., Mann M.K., Svensson P., Sessle B.J., Arendt-Nielsen L., McErlane K.M. (2007). Systemic administration of monosodium glutamate elevates intramuscular glutamate levels and sensitizes rat masseter muscle afferent fibers. Pain.

[22] Lee K.H., Blaha C.D. (2006). Apparatus and Method for Modulating Neurochemical Levels in the Brain. U.S. Patent.

[23] Tseng T.T.C., Chang C.-F., Chan W.-C. (2014). Fabrication of implantable, enzyme-immobilized glutamate sensors for the monitoring of glutamate concentration changes *in vitro* and *in vivo*. Molecules.

[24] Doong R.-A., Shih H.-M. (2006). Glutamate optical biosensor based on the immobilization of glutamate dehydrogenase in titanium dioxide sol-gel matrix. Biosens. Bioelectron..

[25] Laiwattanapaisal W., Yakovleva J., Bengtsson M., Laurell T., Wiyakrutta S., Meevootisom V., Chailapakul O., Emneus J. (2009). On-chip microfluidic systems for determination of L-glutamate based on enzymatic recycling of substrate. Biomicrofluidics.

[26] Ligler F.S., Rowe Taitt C.A. (2002). Optical Biosensors—Present & Future.

[27] Qin S., van der Zeyden M., Oldenziel W., Cremers T., Westerink B. (2008). Microsensors for *in vivo* measurement of glutamate in brain tissue. Sensors.

[28] Maalouf R., Chebib H., Saïkali Y., Vittori O., Sigaud M., Jaffrezic-Renault N. (2007). Amperometric and impedimetric characterization of a glutamate biosensor based on Nafion^®^ and a methyl viologen modified glassy carbon electrode. Biosens. Bioelectron..

[29] Muehlbauer M.J., Guilbeau E.J., Towe B.C., Brandon T.A. (1990). Thermoelectric enzyme sensor for measuring blood glucose. Biosens. Bioelectron..

[30] Muehlbauer M.J., Guilbeau E.J., Towe B.C. (1990). Applications and stability of a thermoelectric enzyme sensor. Sens. Actuator B. Chem..

[31] Zhang Y., Tadigadapa S. (2004). Calorimetric biosensors with integrated microfluidic channels. Biosens. Bioelectron..

[32] Lee W., Fon W., Axelrod B.W., Roukes M.L. (2009). High-sensitivity microfluidic calorimeters for biological and chemical applications. Proc. Nat. Acad. Sci. USA.

[33] Kopparthy V.L., Tangutooru S.M., Nestorova G.G., Guilbeau E.J. (2012). Thermoelectric microfluidic sensor for bio-chemical applications. Sens. Actuator B. Chem..

[34] Lvov Y., Ariga K., Ichinose I., Kunitake T. (2002). Assembly of multicomponent protein films by means of electrostatic layer-by-layer adsorption. J. Am. Chem. Soc..

[35] Miller S.L., Smith-Magowan D. (1990). The thermodynamics of the Krebs cycle and related compounds. J. Phys. Chem. Ref. Data.

[36] Okumura W., Moridera N., Kanazawa E., Shoji A., Hirano-Iwata A., Sugawara M. (2009). Visualizing L-glutamate fluxes in acute hippocampal slices with glutamate oxidase-immobilized coverslips. Anal. Biochem..

